# A comparative study in elective repair of large incisional hernias using on-lay mesh vs. sub-lay mesh: a meta-analysis

**DOI:** 10.1007/s13304-024-01755-0

**Published:** 2024-02-19

**Authors:** Basma Hussein Abdelaziz Hassan, Kirollos Adel Louiz Kamel, Philobater Bahgat Adly Awad, Kerolos Bahgat Adly Awad, Sameh Abdallah Maaty, Fawzi Salah Fawzi, Bassem Helmy El-Shayeb

**Affiliations:** General Surgery Department, Faculty of Medicine, Ain Shams University, Cairo, Egypt

**Keywords:** Incisional hernia, Sub-lay mesh on-lay mesh, Retromuscular mesh, Polypropylene

## Abstract

Ventral abdominal wall incisional hernia is defined as a defect in the musculo-fascial layers of the abdominal wall in the region of the postoperative scar. There is a slight increase in the incidence of incisional hernia in the female gender. The higher percentage of incisional hernia in females might be due to laxity of abdominal wall muscles after multiple pregnancies and also an increased incidence of obesity in females. To assess incisional hernia repair using two different techniques: on-lay mesh and sub-lay mesh, as regards operative time, postoperative recurrence, wound infection, seroma, hematoma, and flap necrosis. Pubmed, Web of Science, and Scopus were searched on 15 March 2022. The keywords incisional hernia, sub-lay mesh on-lay mesh, retromuscular mesh, and polypropylene. According to our results, there is a statistical difference between onlay and sublay regarding intra-operative time as sublay mesh is more time-consuming. Regarding postoperative complications, there is no statistical difference in recurrence, seroma, hematoma, flap necrosis, and infection but there is a statistical difference regarding in hospital stay as patients with sub-lay repair stays less than only.

## Introduction

Ventral abdominal wall incisional hernia is defined as a defect in the musculo-fascial layers of the abdominal wall in the region of postoperative scar [1].

There is a slight increase in the incidence of incisional hernia in the female gender. The higher percentage of incisional hernia in females might be due to laxity of abdominal wall muscles after multiple pregnancies and also an increased incidence of obesity in females [2].

The most common risk factor for the development of incisional hernia was the occurrence of wound infection after the previous surgery which was found in 46.67% of their cases. Also, obesity, smoking, chronic cough, diabetes mellitus, and anemia were important risk factors for incisional hernia development [3].

Incisional hernia repair can be done by either an open or a laparoscopic technique. The open technique can be a simple suture repair or a mesh repair [4].

The mesh fixation technique is the gold standard procedure for incisional hernia repair. Restriction to the principles of repair reduces the postoperative complications and recurrence rates. These principles include: strict aseptic technique, tension-free repair, repair of the whole previous surgical scar, closure of the fascial defect with non-absorbable sutures taking good bites with narrow intervals, making at least 5 cm mesh overlap of the hernial defect in all directions, and prophylactic use of antibiotics post-operatively [5].

Open mesh repair is the standard procedure for incisional hernia repair. The mesh can be placed between the subcutaneous tissues of the abdominal wall and the anterior rectus sheath (on-lay repair) as well as it can be placed in the pre-peritoneal space or the retro-muscular space created between the rectus muscle and posterior rectus sheath (sub-lay repair) [6].

The mean operative time in the sub-lay technique is more than the mean operative time in the on-lay technique due to the time consumed in dissecting the retro-rectal or pre-peritoneal space [7].

Seroma formation after drain removal is a common complication after incisional hernia repair. In many previous studies, the rate of seroma formation after the on-lay repair is much more than that after the sub-lay repair with statistically significant distribution [8].

Studies have shown that 70–75% of incisional hernia recurrences develop within 2 years and 80–90% develop within 3 years [9].

### Aim of the work

This study aims to assess incisional hernia repair using two different techniques: on-lay mesh and sub-lay mesh, as regards operative time, postoperative recurrence, wound infection, seroma, hematoma, and flap necrosis.

## Patients and methods

### Search strategy

**Fig. 1:**
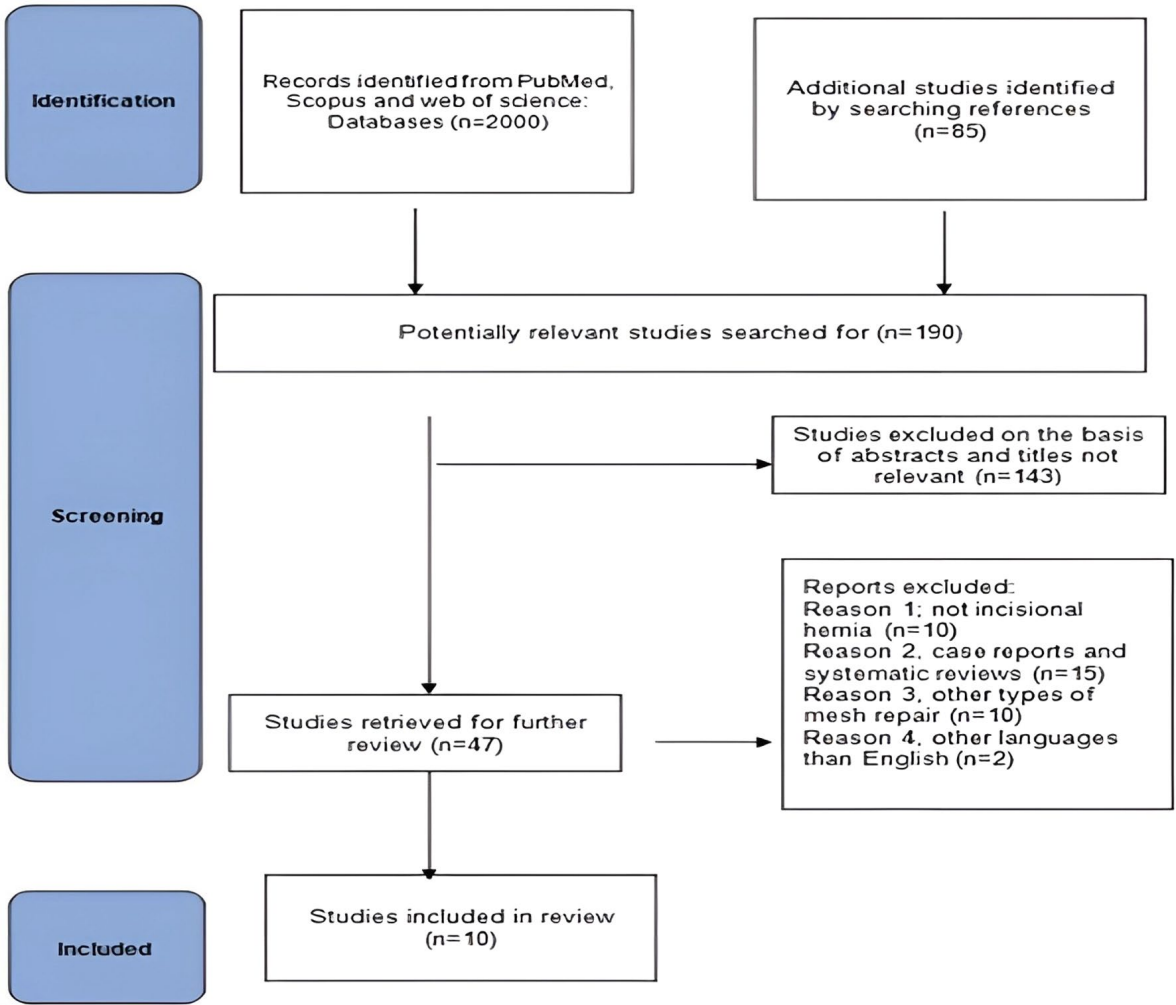
Shows PRISMA flowchart

Pubmed, Web of Science, and Scopus were searched on 15 March 2022. The keywords *incisional hernia, sub-lay mesh on-lay mesh, retromuscular mesh, and polypropylene.* The details of the search process and study selection are shown in Figure (42)*.* Relevant articles referenced in these primary studies were also searched to enroll additional cases, some articles were searched from the references of some studies (Fig. 1).

### Eligibility criteria

All trials about the use include participants who are diagnosed with large incisional hernia and above 18 years. Studies designed for comparing on-lay mesh and sub-lay mesh randomized controlled trials and prospective or retrospective cohort studies were included, provide sufficient details on the above outcome measures to allow comparison across studies and report quantitative data in addition to, published in full-text and a peer-reviewed journal. Patients who were unfit for general anesthesia and refused surgery, in addition to papers in other languages than English, and reviews, case reports, or studies regarding animals were excluded.

### Outcomes

Outcomes of this study included operative time, hospital stay postoperative recurrence, wound infection, seroma, hematoma, and flap necrosis. 

### Quality assessment

Each article was assessed by two independent researchers based on the Cochrane Handbook 5.0.2 and data were extracted separately by the two researchers. The included trials were evaluated with the following criteria: adequate sequence generation, allocation concealment, blinding of participants and outcome assessors, incomplete outcome data, free of selective reporting, and free of other biases. Each type of bias was defined by an answer (Yes/No/Unclear). “Yes” indicated a low risk of bias, “No” represented a high risk, and “Unclear” represented an unclear risk. In addition, the quality of evidence for each outcome was assessed by the Grading of Recommendations, Assessment, Development, and Evaluations (GRADE) system (33) [10].

Characteristics and quality assessment of enrolled studies are listed in Tables 1 and 2

**Table 1:** Shown quality assessment in cohort studies

	Selection				Comparability	Outcome			Results
Study	Is the case definition adequate?	Selection of controls	Representativeness of the cases	Definition of controls	Comparability of cases and controls on the basis of the design or analysis	Assessment of outcome	Was follow-up long enough for outcomes to occur	Adequacy of follow-up of cohorts	Good\Fair\Poor
Soomro 2018 [11]	Yes		Yes		Yes	Yes	Yes	Yes	(6) Fair
Saeed 2013 [12]	Yes	Yes	Yes	Yes	Yes	Yes	Yes	Yes	(8) Good
El Badawy 2020 [13]	Yes		Yes	Yes		Yes	Yes	Yes	(6) Fair
Leithy 2013 [14]	Yes		Yes			Yes	Yes	Yes	(5) Fair
Deen 2018 [15]	Yes		Yes	Yes		Yes	Yes	Yes	(6) Fair
A. Iljin 2019 [16]	Yes		Yes			Yes	Yes	Yes	(5) Fair

**Table 2:** Showing quality assessment in randomized controlled trials studies

Study	Adequate sequence generation	Allocation concealment	Blinding	Incomplete outcome data	Free of selective reporting	Free of other bias
Zaza Demetrashvili 2016 [17]	Yes	Yes	Yes	Yes	Unclear	Unclear
Ahmed 2017 [18]	Yes	Yes	Yes	Yes	Yes	Unclear
Barış Sevinç 2018 [19]	Yes	Yes	Yes	Yes	Unclear	Unclear
S. Natarajan 2017 [20]	Yes	Yes	Yes	Yes	Yes	Unclear

### Statistical analysis

Statistical analysis was performed by Open Metaanalyst version 5.3 software. The odds ratio (OR) with a 95% confidence interval (95% CI) for dichotomous variables and the mean difference (MD) with 95% CI for continuous variables were computed in the fixed effect or random-effect model. Heterogeneity among trials was justified using a chi-squared test with *P* < 0.1 demonstrating statistical significance. The quantity of heterogeneity was measured by *I*2 and *I*2 > 50% indicated significant heterogeneity. If no significant heterogeneity was confirmed, we did the meta-analysis in a fixed effect model. Or else, the random-effect model was used.

## Results

Ten trials included, regarding the comparison between onlay and sub-lay mesh in large incisional hernia repair were selected from electronic databases [1, 11–14, 16–20] (Table 3).

**Table 3:** Outcome parameters: 1: recurrence 2: hospital stay 3: operative time 4: hematoma 5: seroma 6: infection 7: flap necrosis

Study	Study design	Sample size	Repair method	FU/month	Outcome
Zaza Demetrashvili 2016 [17]	A randomized controlled trial	180	Onlay vs sublay	72 ms	1, 2, 3, 4, 5, 6
Ahmed 2017 [18]	A randomized controlled trial	65	Onlay vs sublay	72 ms	1, 2, 3, 5, 6
Soomro 2018 [11]	Prospective cohort	200	Onlay vs sublay	24 ms	1, 2, 5, 6, 7
Saeed 2013 [12]	Prospective cohort	80	Onlay vs sublay	24 ms	1, 2, 3, 4, 5, 6
Barış Sevinç 2018 [19]	A randomized controlled trial	100	Onlay vs sublay	46 ms	2, 3, 4, 5, 6
EL BADAWY 2020 [13]	Prospective cohort study	120	Onlay vs sublay	24 ms	1, 2, 3, 5, 6
Leithy 2013 [14]	Prospective cohort study	30	Onlay vs sublay	12 ms	1, 5, 6, 7
Deen 2018 [15]	Prospective cohort study	40	Onlay vs sublay	12 ms	1, 2, 3, 4, 5
S. Natarajan 2017 [20]	Randomized controlled trial	24	Onlay vs sublay	6 ms	1, 5, 6
A. Iljin 2019 [16]	Retrospective cohort	40	Onlay vs sublay	72 ms	1, 2, 3, 4, 5, 6, 7

### Recurrence

**Fig. 2:**
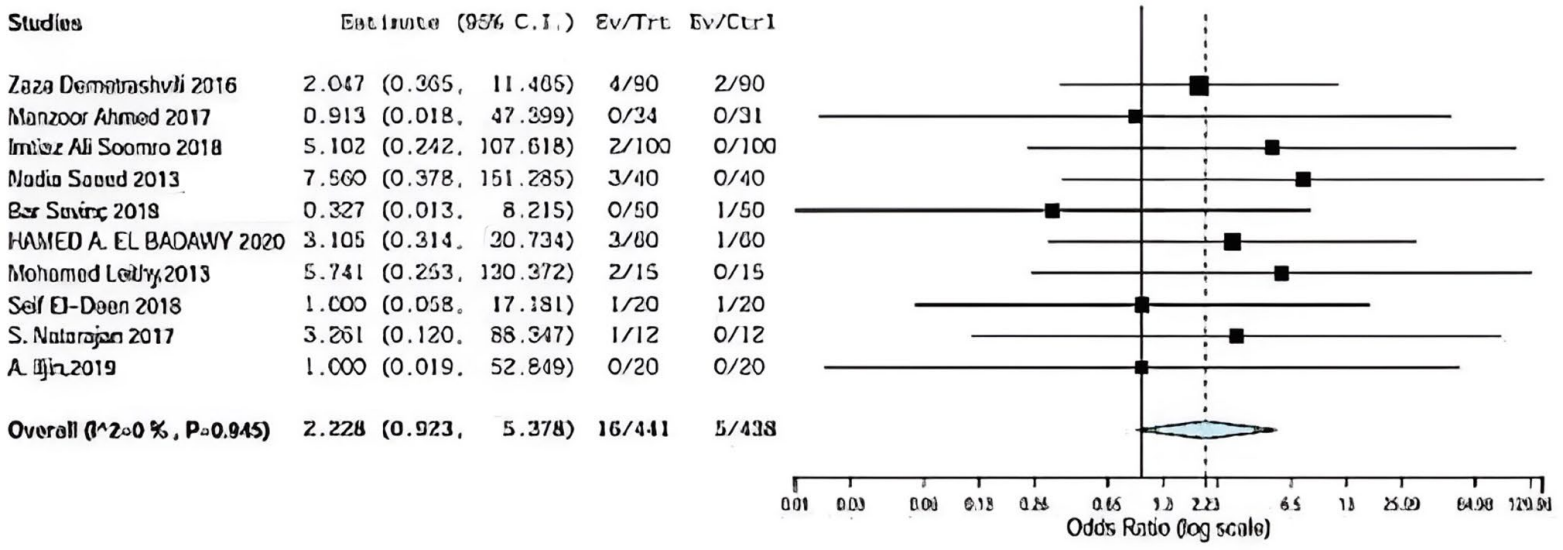
Shows recurrence in onlay versus sublay

Ten included studies described recurrence using onlay versus sub-lay with follow-up at least 3 months, Demetrashvili et al. [17], Ahmed et al. [18], Somooro et al. [11], Saeed et al. [12], Barış Sevinç et al. [19]., Badawy et al. [13] in addition to, Leithy et al. [14], Deen et al. [15] S. Natarajan et al. [20] and A. Iljin et al. [16] with (*P* = 0.94, *I*2 = 0%) and OR 2.228, 95% CI 0.9, 5.378 and no statistical significance Fig. 2.

### Infection

**Fig. 3:**
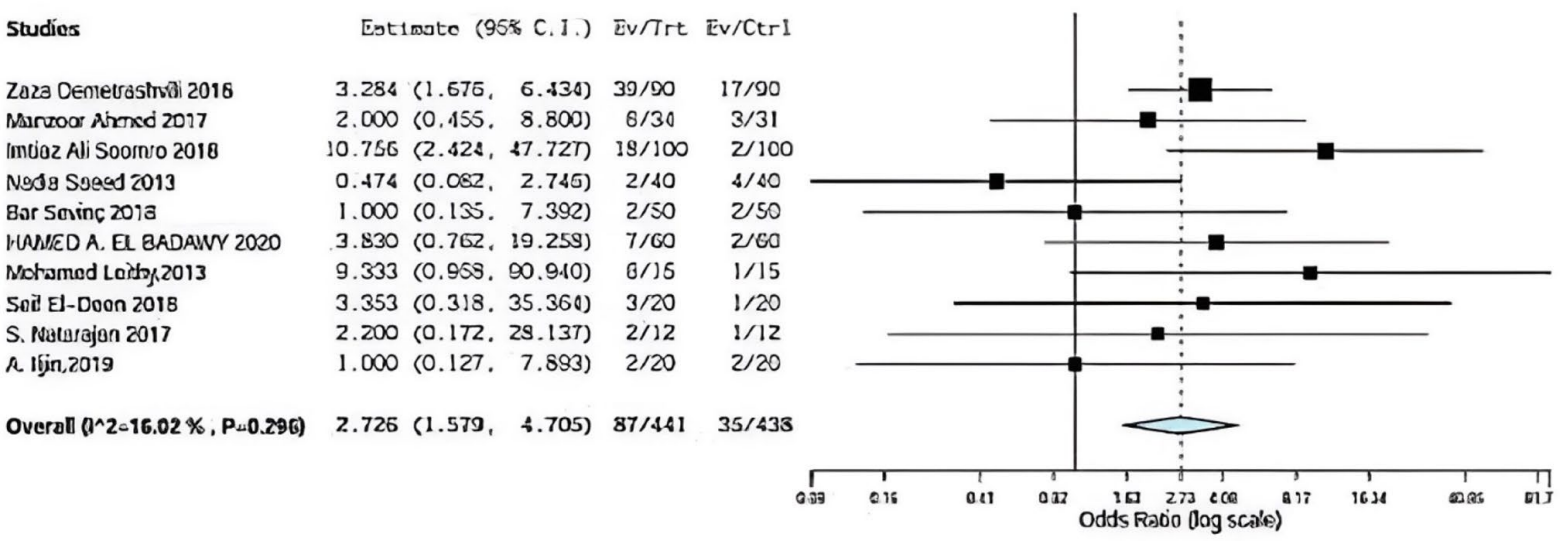
Shows infection in onlay versus sublay

Ten included studies described infection using onlay versus sublay with a follow-up at least 3 months, Demetrashvili et al. [17], Ahmed et al. [18], Somooro et al. [11], Saeed et al. [12], Barış Sevinç et al. [19], in addition to, Leithy et al. [13], Deen et al. [15]. S. Natarajan et al. [20] and A. Iljin et al. [16] with (*P* = 0.296, *I*2 = 16%) and OR 2.726, 95% CI 1.579, 4.705 and no statistical significance Fig. 3.

### Seroma

**Fig. 4:**
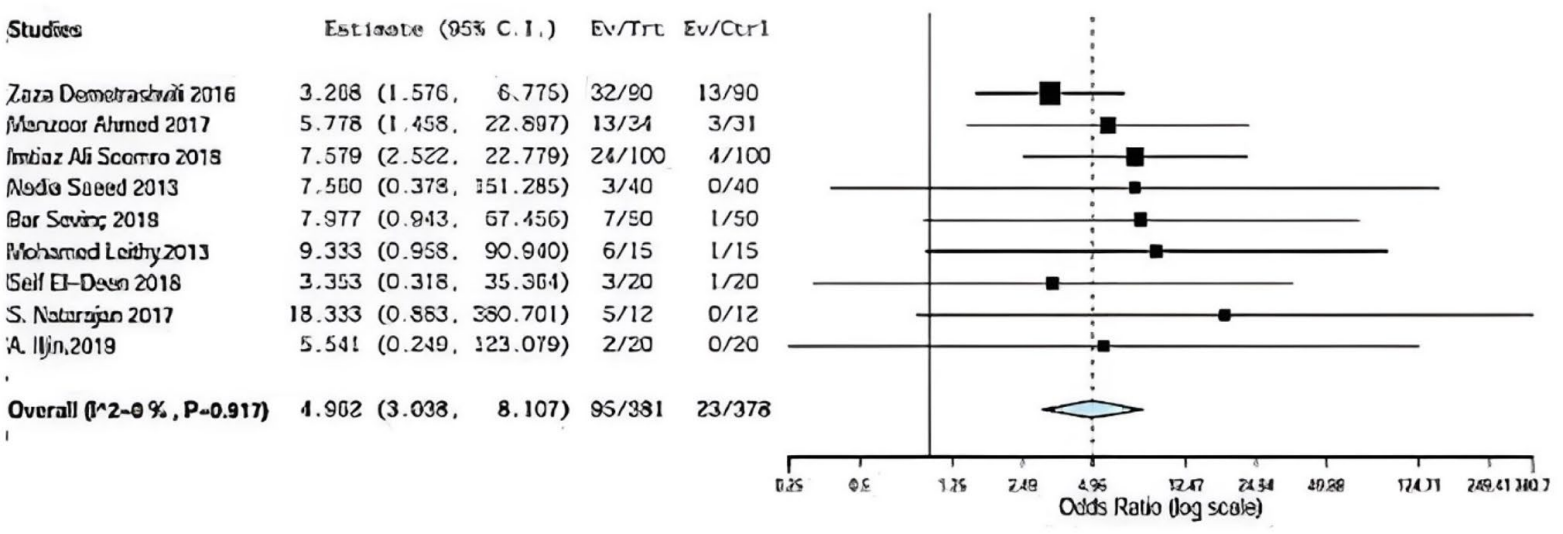
Shows seroma in onlay versus sublay

Seroma was assessed in nine included studies comparing between onlay versus sublay. Demetrashvili et al. [17], Ahmed et al. [18], somooro et al. [11], Saeed et al. [12], Barış Sevinç et al. [19], Badawy et al. [13] in addition to, S. Natarajan et al. [20] and A. Iljin et al. [16] with (*P* = 0.917, *I*2 = 0%) and OR 4.962, 95% CI 3.038, 8.107 and no statistical significance Fig. 4.

### Hematoma

Hematoma was assessed in four included studies comparing onlay versus sublay.

**Fig. 5:**
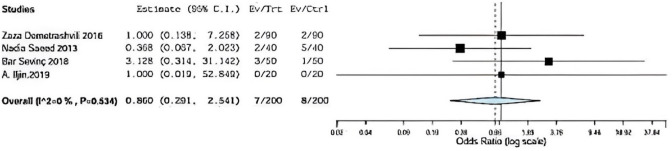
Shows hematoma in onlay versus sub-lay

Demetrashvili et al. [17], Saeed et al. [12], Barış Sevinç et al. [19], in addition to A. Iljin [16] et al. with (*P* = 0.534, *I*2 = 0%) and OR 0.860, 95% CI 0.291, 2.541 and no statistical significance Fig. 5.

### Flap necrosis

**Fig. 6:**
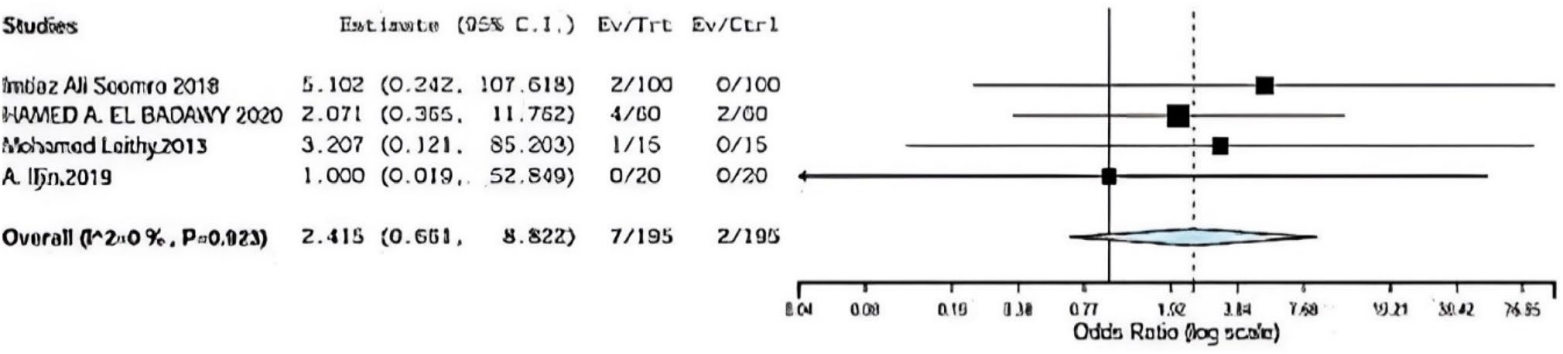
Shows flap necrosis in onlay versus sublay

Flap necrosis was assessed in four included studies comparing onlay versus sub-lay with follow-up at least 3 months, Somooro [11] et al., Badawy et al. [13] in addition to, Leithy et al. [14], and A. Iljin et al. [16] with (*P* = 0.923, *I*2 = 0%) and OR 2.415, 95% CI 0.661, 8.822 and no statistical significance Fig. 6.

### Operative time

**Fig. 7:**
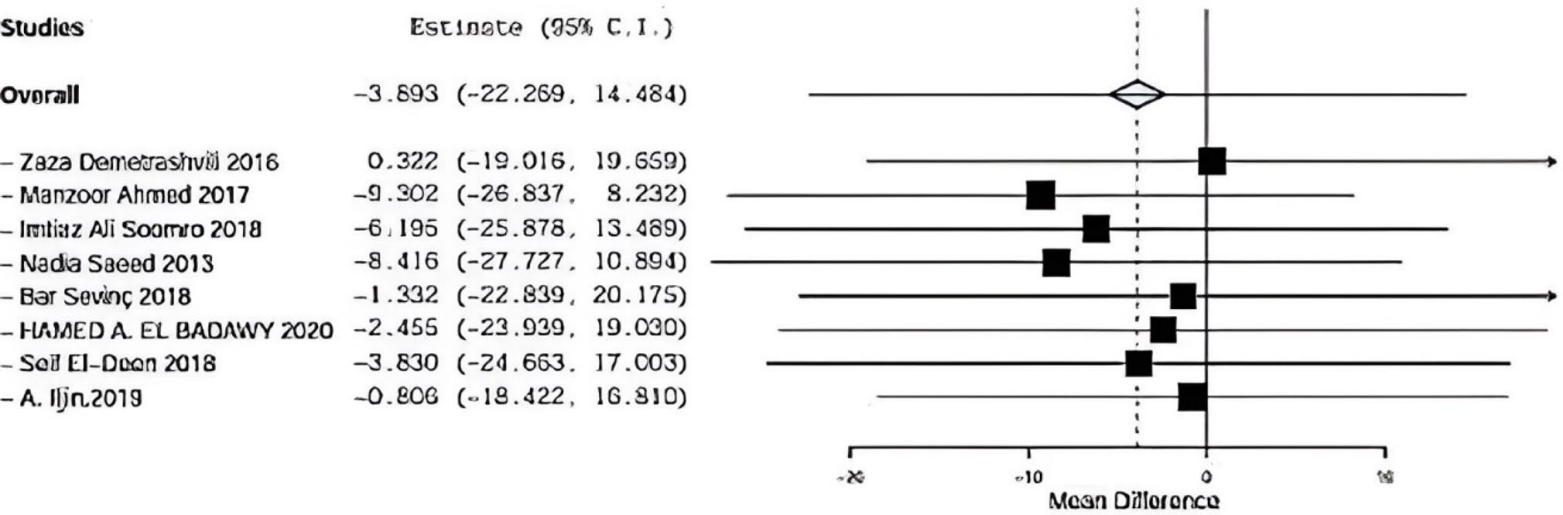
Shows operative time in onlay versus sublay

Eight included studies described operative time using onlay versus sublay, Zaza Demetrashvili et al. [17], Ahmed et al. [18], Soomro et al. [11], Saeed et al., Barış Sevinç et al. [19], Hamed et al. in addition to, Seif et al. and A. Iljin et al. with (*P* = 0.001, *I*2 = 95.1%) and OR 12.022, 95% CI 31,460, 5.616 and there is statistical significance Fig. 7.

### Hospital stay

**Fig. 8:**
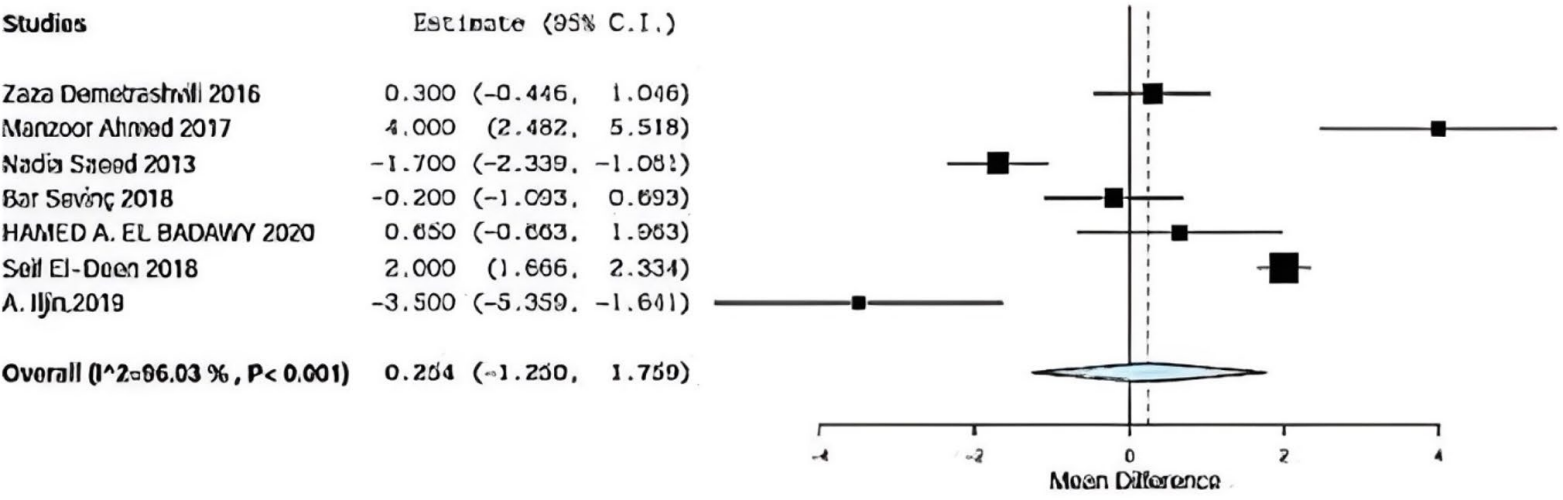
Shows hospital stay in onlay versus sublay

Seven included studies described hospital stay using onlay versus sublay, Demetrashvili et al. [17], Ahmed et al. [18], Saeed et al. [12], Barış Sevinç et al. [19], Badawy et al. [13] in addition to, Deen et al. [15] and A. Iljin [16] et al. with (*P* = 0.001, *I*2 = 96.03%) and OR 2.726, 95% CI 1.250, 1.759 with statistically significance Fig. 8.

### Sensitivity of each of the outcomes

#### Seroma sensitivity

**Fig. 9:**
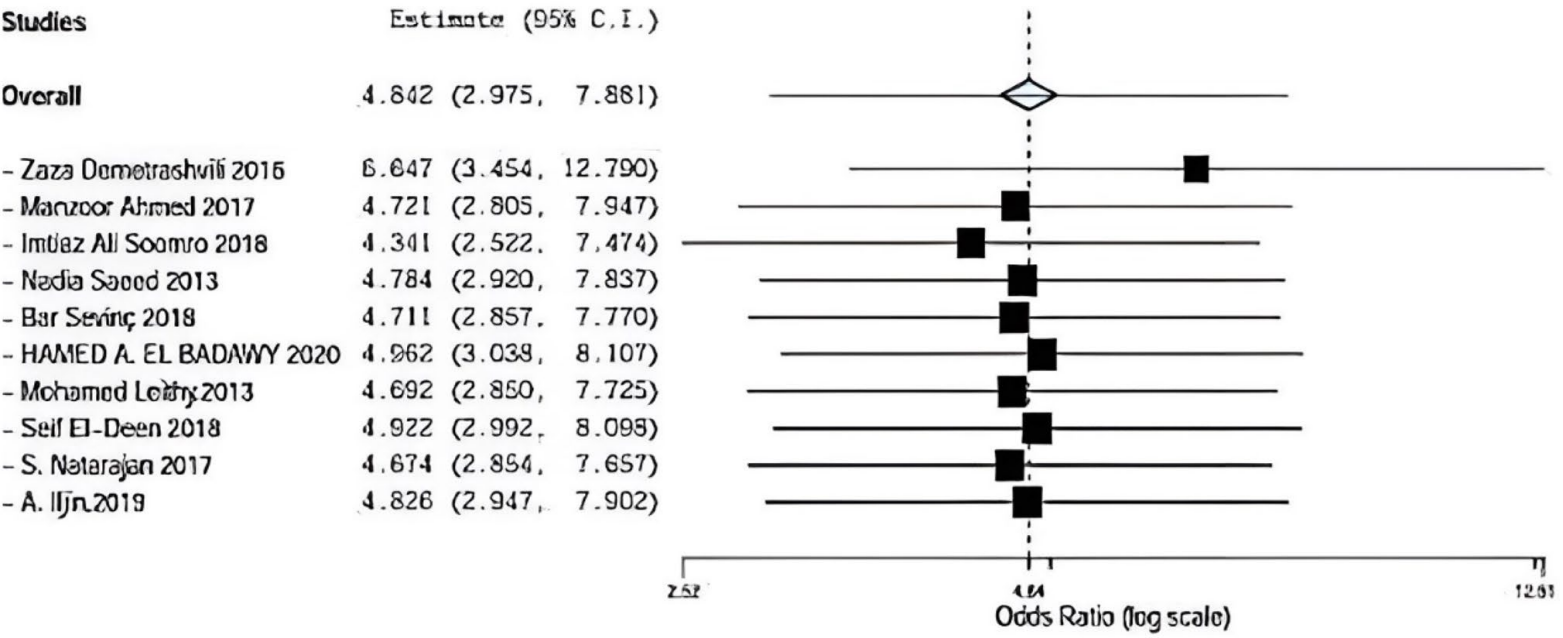
Sensitivity

Regarding sensitivity in seroma in nine trials, the overall effect is 4.729. By removing Demetrashvli et al. [17], study overall effect is 6.640, and by removing Ahmed et al. [18], study overall effect is 4.585. By removing Somooro et al. [11], overall effect is 4.172, and by removing Baris sevinc [19], overall effect is 4.586. Similarly, by removing Leithy et al. [14], overall effect is 4.566. By removing Deen et al., overall effect is 4.808, by removing A. ILjin [16], overall effect is 4.709, and there was a deviation in the result when leaving one paper out as seen in Fig. 9.

### Recurrence sensitivity

**Fig. 10:**
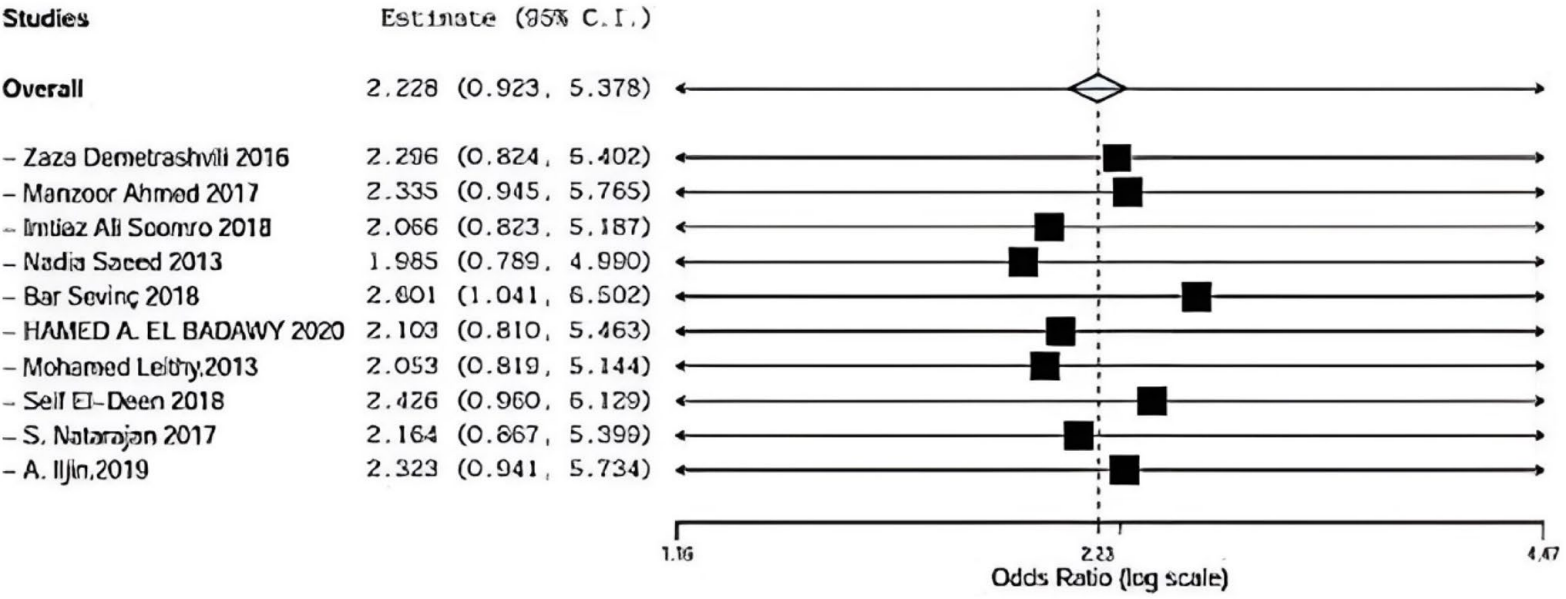
Shows sensitivity in recurrence

Regarding sensitivity in recurrence in ten trials, the overall effect is 2.228. By removing Demetrashvli et al. [17], study overall effect is 2.296, and by removing Ahmed et al. [18] study overall effect is 2.335, and by removing Somooro et al. [11], overall effect is 2.066. By removing Saeed et al. [12], the overall effect is 1.985, Bar Sevinc et al. [19]. The overall effect is 2.601, by removing Badawy et al. overall effect is 2.103 Leithy et al. [14]. Overall effect is 2.053, by removing Deen et al. [15].overall effect is 2.426, by removing S. Natarajan [20] overall effect is 2.164 and by removing A. ILjin [16] overall effect is 2.323 there was no deviation in the result when leaving one paper out as seen in Fig. 10

#### Hematoma sensitivity

**Fig. 11:**
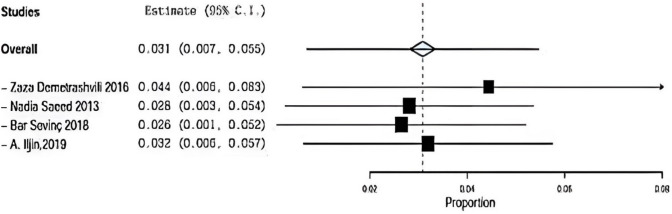
Shows sensitivity in hematoma

Regarding sensitivity in hematoma four trials, the overall effect is 0.031. By removing Demetrashvli et al. [17], study overall effect is 0.044, and by removing Saeed et al. [12], overall effect is 0.028. Similarly for Bar sevinc [19] et al., overall effect is 0.026 and by removing A. ILjin [16], overall effect is 0.032, and there was a deviation in the result when leaving one paper out as seen in Fig. 11.

#### Flap necrosis Sensitivity

**Fig. 12:**
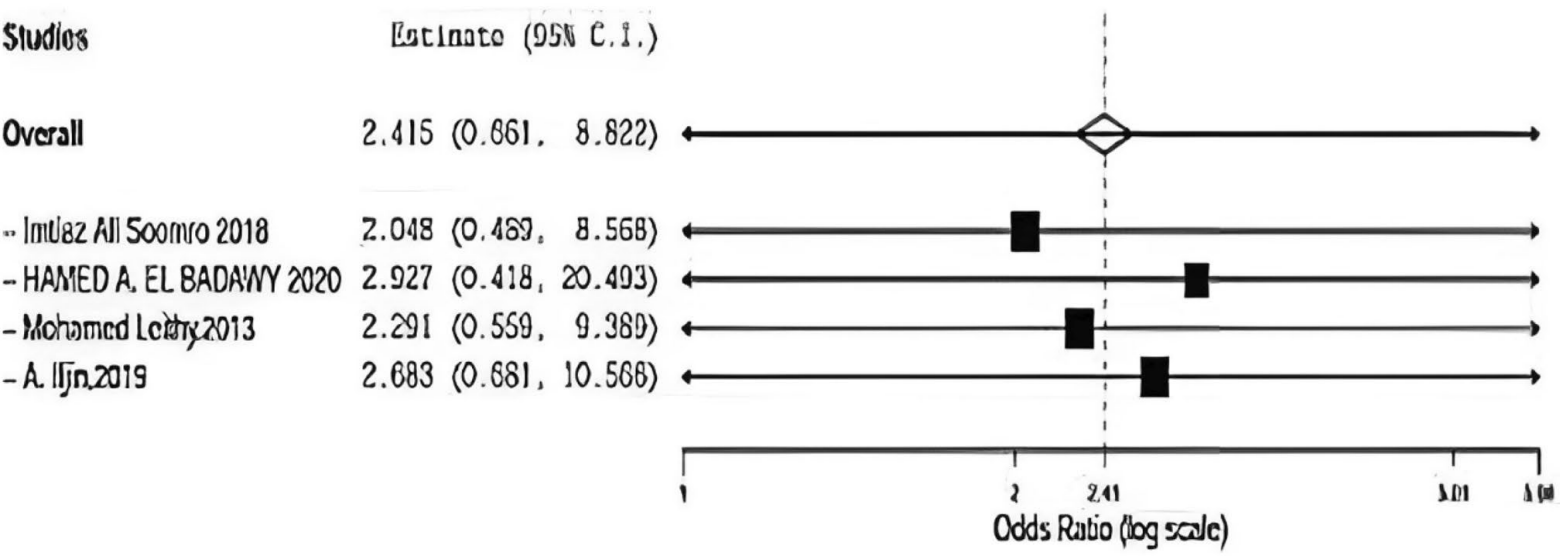
Shown sensitivity in flap necrosis

Regarding sensitivity in flap necrosis in four trials, the overall effect is 2.415. By removing Saeed et al [12], overall effect is 2.048, and by removing Badawy et al. [13], the overall effect is 2.927. By removing Leithy et al. [14], overall effect is 2.291, and by removing A.ILjin [16] overall effect is 2.683, and there was no deviation in the result when leaving one paper out as seen in Fig. 12.

#### Operative time sensitvity

**Fig. 13:**
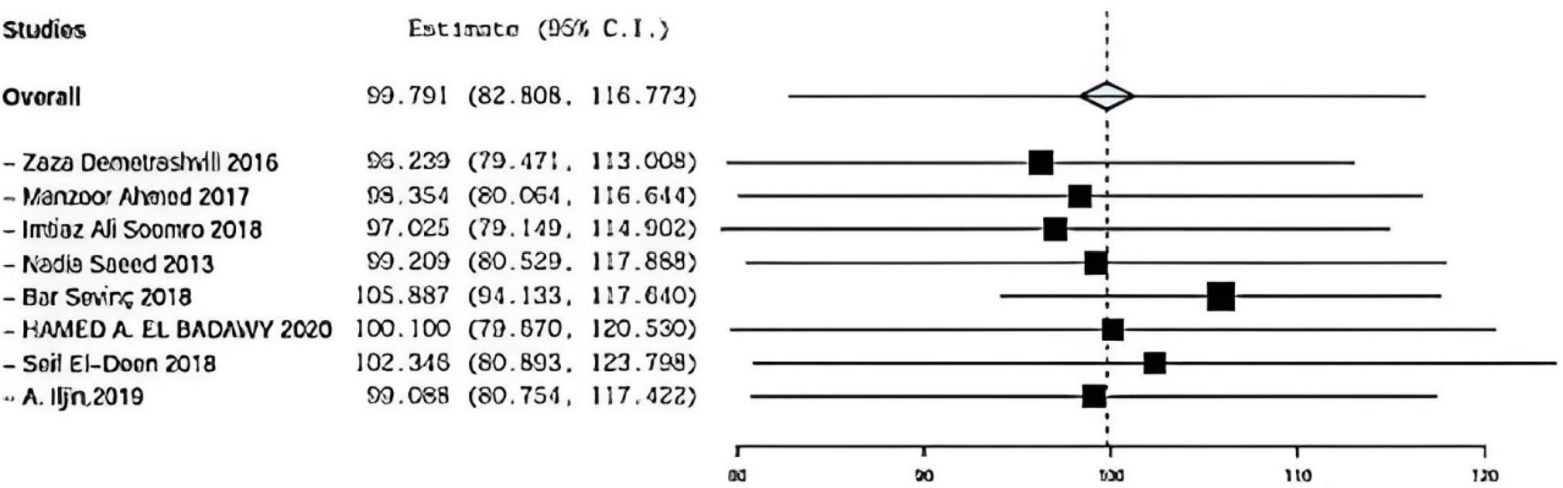
operative time sensitivity

Regarding sensitivity in operative time in eight trials, the overall effect is 99.7. By removing Demetrashvli et al study, overall effect is 96.23, and by removing Ahmed et al. [18], study overall effect is 98.35. By removing Somooro et al. [11], overall effect is 97.025, and by removing Saeed et al., overall effect is 99.209. Similarly, for Bar sevinc et al. [19], overall effect is 105.88, and by removing Badawy et al. [13], overall effect is 100. By removing Deen et al. [15], overall effect is 102.346 and by removing A.ILjin [16] overall effect is 99.088, and there was no deviation in the result when leaving one paper out as seen in Fig. 13.

#### Hospital Stay Sensitivty

Regarding sensitivity in hospital stay in seven trials, the overall effect is 0.254. By removing Demetrashvli et al. [17], study overall effect is 2.239, and by removing Ahmed et al. [18] study, overall effect is − 0.322. By removing Saeed et al. [12], overall effect is 0.625. Similarly, Bar sevinc et al. [19], overall effect is 0.328. By removing Badawy et al. [13], overall effect is 0.188, and by removing Deen et al. [15], overall effect is − 0.063.

**Fig. 14:**
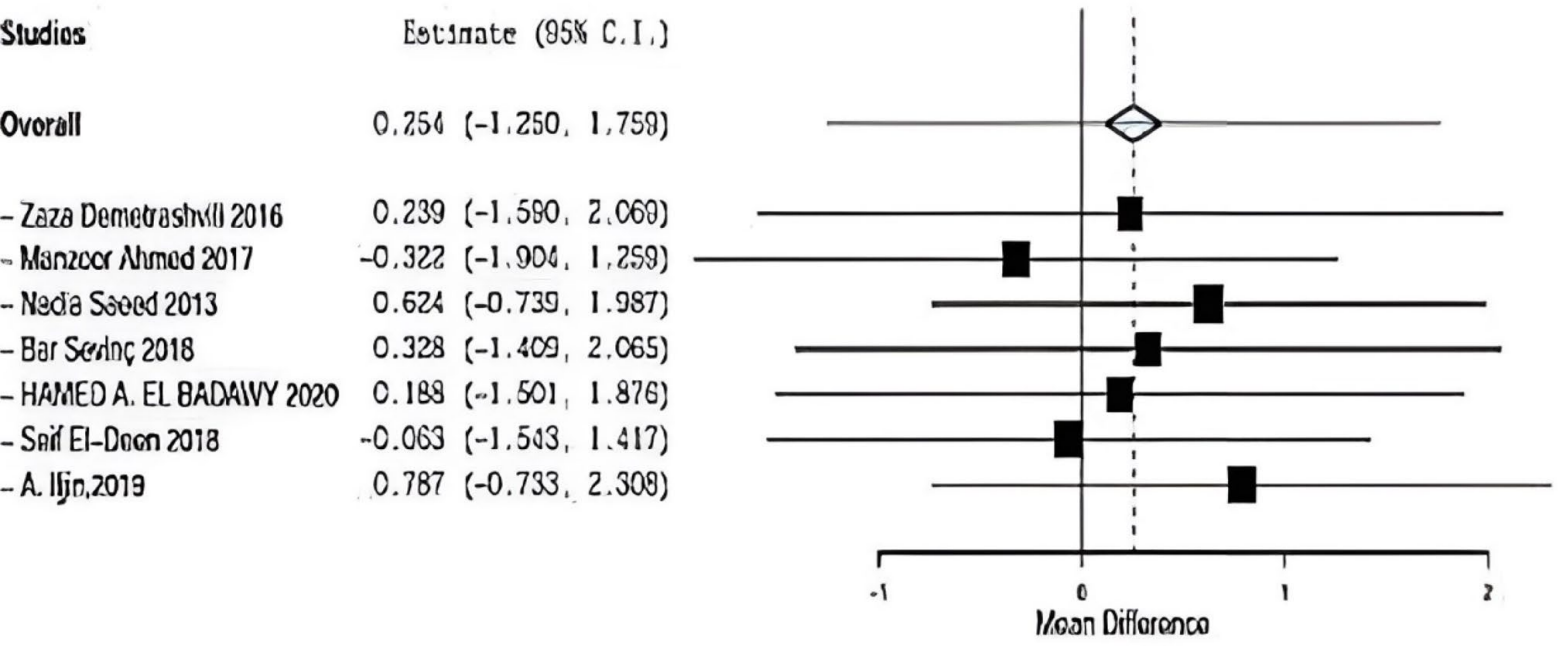
Shows sensitivity in hospital stay

By removing A.ILjin [16], overall effect is 0.787, and there was no deviation on the result when leaving one paper out as seen in Fig. 14.

#### Infection Sensitivty

**Fig. 15:**
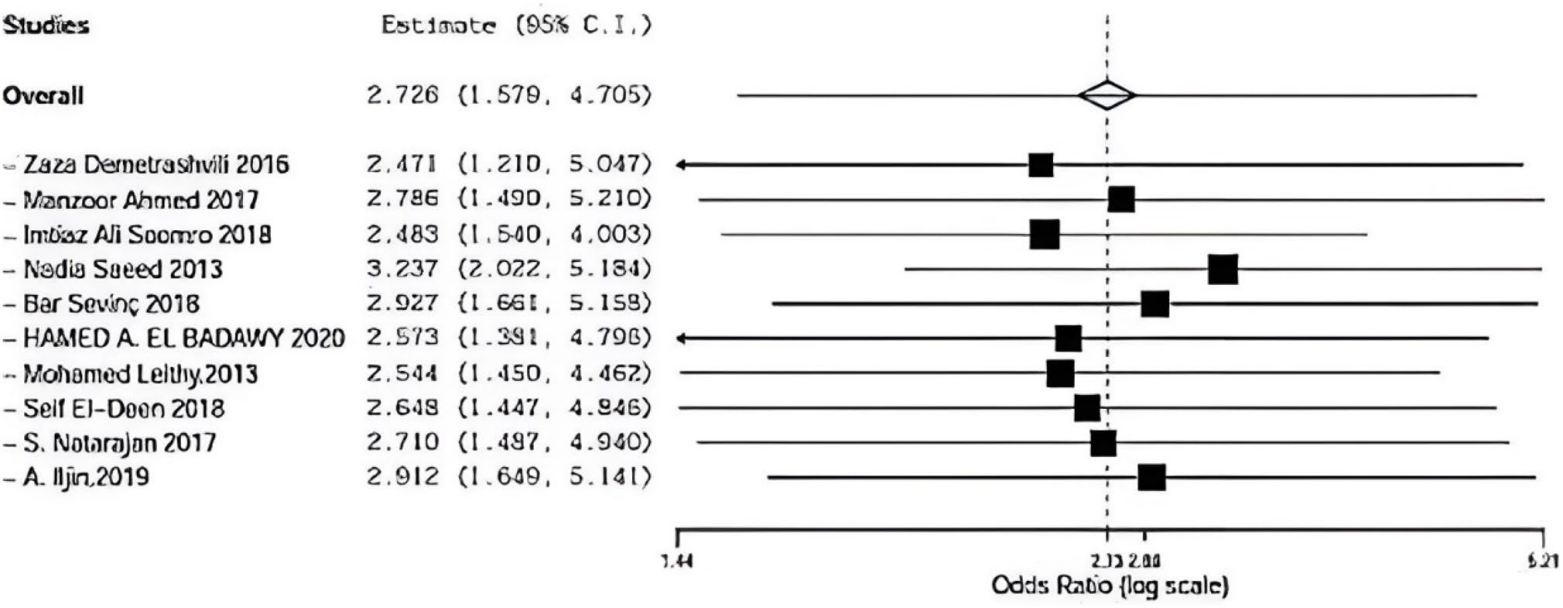
Infection

Regarding infection in ten trials, the overall effect is 2.726. By removing Demetrashvli et al. [17] study, overall effect is 2.471, and by removing Ahmed et al. [18] study, overall effect is 2.786. By removing Somooro et al. [11], overall effect is 2.483, and by removing Saeed et al. [14], overall effect is 3.237. Similarly, for Bar sevinc et al. [19], overall effect is 2.927. By removing Badawy et al. [13], overall effect is 2.573. For Leithy et al. [14], overall effect is 2.544, and by removing Deen et al. [15], overall effect is 2.648. By removing S. Natarajan [20], overall effect is 2.710 and by removing A. ILjin [16], overall effect is 2.912, and there was no deviation in the result when leaving one paper out as seen in Fig. 15.

## Discussion

The current meta-analysis is to compare intra-operative difficulties and postoperative complications in elective repair of large incisional hernias using on-lay mesh and sub-lay mesh. We have comprehensively searched and assessed the published literature regarding this topic. We have focused solely on the data regarding the comparison between on-lay mesh and sub-lay mesh using the random-effects model.

Repair of incisional hernia is regarded as one of the most challenging general surgical procedures, due to the high recurrence rate and post-operative morbidity. Open mesh repair (onlay and sublay technique) is proved to be superior to suture repair. However, due to the presence of mesh, this technique is not without morbidity such as wound complications like seroma formation and infection [21].

In our study, ten trials included a comparison between onlay and sub-lay mesh in large incisional hernia repair were selected from electronic databases.

As regards recurrence, ten included studies described recurrence using onlay versus sublay with a follow-up of at least 3 months, Demetrashvili et al. [17] stated 4 patients suffered from a recurrence in 72 months of follow-up using the onlay method and 2 patients using sub-lay method. Ahmed R et al. stated zero patients suffered from a recurrence in 72 months of follow-up using the onlay method and also zero patients using the sublay method. Somooro et al. stated 2 patients suffered from a recurrence in 24 months follow-up using onlay method and zero patients using sub-lay method.

Saeed et al. [12] stated 3 patients suffered from a recurrence in 24-month follow-up using the onlay method and zero patients using sub-lay method. Barış Sevinç et al. [19] stated zero patients suffered from recurrence in 46 months of follow-up using on-lay method and one patient using sub-lay method. Badawy et al. [13] stated three patients suffered from recurrence in 24 months of follow-up using on-lay method and one patient using the sub-lay method.

In addition, Leithy et al. [14] stated that 2 patients suffered from recurrence in 12 months of follow-up using the onlay method and zero patients using the sublay method. Deen et al. [15] stated one patient suffered from recurrence in 12 months of follow-up using the onlay method and one patient using sub-lay method. S. Natarajan et al. [20] stated one patient suffered from a recurrence in 6 months of follow-up using the onlay method and zero patients using sub-lay method, and A. Iljin et al. [16] stated zero patients suffered from a recurrence in 72 months of follow-up using the onlay method and zero patients using sublay method (*P* = 0.94, *I*2 = 0%) and OR 2.228, 95% CI 0.9, 5.378 and no statistical significance.

Regarding infection, ten included studies described infection using onlay versus sublay with follow-up at least 3 months. Demetrashvili et al. [17] stated that 39 patients in 72 months followed using onlay method and 17 patients using sublay method. Ahmed et al. stated 6 patients in 72-month follow-up using onlay method and also 3 patients using sublay method. Somooro et al. [11] stated 18 patients in 24-month follow-up using onlay method and 2 patients using the sublay method, and Saeed et al. [12] stated 2 patients in 24-month follow-up using the onlay method and 4 patients using the sublay method. Barış Sevinç et al. [19] stated 2 patients in 46-month follow-up using onlay method and 2 patients using sublay method. Badawy et al. stated 7 patients in 24-month follow-up using onlay method and 2 patients using sublay method. In addition, Leithy et al. [14] stated 6 patients in 12-month follow-up using onlay method and 1 patient using the sublay method. Deen et al. [15] stated 3 patients in 12-month follow-up using onlay method and one patient using sub-lay method. S. Natarajan et al. [20] stated 2 patients in 6-month follow-up using the onlay method and 1 patient using the sublay method.

A. Iljin et al. [16] stated 2 patients in 72-month follow-up using the onlay method and 2 patients using sub-lay method (*P* = 0.296, *I*2 = 16%) and OR 2.726, 95% CI 1.579, 4.705 and no statistical significance.

In addition, seroma was assessed in nine included studies comparing between onlay versus sublay.

Demetrashvili et al. [17] stated 32 patients in 72 months of follow-up using onlay method and 13 patients using the sublay method. Ahmed et al. [18] stated 13 patients in 72 months of follow-up using the onlay method and also 3 patients using sublay method. Somooro et al. [11] stated 24 patients in 24 months of follow-up using the onlay method and 4 patients using the sublay method, and Saeed et al. [12] stated 3 patients in 24 months of follow-up using the onlay method and zero patient using sublay method. Barış Sevinç et al. [19] stated 7 patients in 46 months follow-up using onlay method and 1 patient using sublay method.

In addition, Leithy et al. [14] stated 6 patients in 12 months of follow-up using onlay method and 1 patient using the sublay method. Deen et al. stated 3 patients in 12 months of follow-up using onlay method and one patient using sublay method. S. Natarajan et al. [20] stated 5 patients in 6 months of follow-up using onlay method and zero patient using sublay method. A. Iljin [16] et al. stated 2 patients in 72-month follow-up using onlay method and zero patient using sublay method (*P* = 0.917, *I*2 = 0%) and OR 4.962, 95% CI 3.038, 8.107 and no statistically significance, while hematoma was assessed in four included studies comparing between onlay versus sublay.

Demetrashvili et al. [17] stated 2 patients using onlay method and 2 patients using sublay method. Saeed et al. [12] stated 2 patients using onlay method and 5 patients using sublay method. Barış Sevinç et al. [19] stated 3 patients using onlay method and 1 patient using sublay method.

In addition, A. Iljin et al. [16] stated zero patient using onlay method and zero patient using sublay method (*P* = 0.534, *I*2 = 0%) and OR 0.860, 95% CI 0.291, 2.541 and no statistically significance.

Flap necrosis was assessed in four included studies comparing between onlay versus sublay.

Somooro et al. [11] stated 2 patients in 24 months of follow-up using onlay method and zero patient using sublay method, and Badawy et al. stated 4 patients in 24 months of follow-up using onlay method and 2 patients using sublay method.

In addition, Leithy et al. [14] stated 1 patient in 12 months of follow-up using onlay method and zero patient using sublay method. A. Iljin et al. [16] stated zero patient in 72 months follow-up using onlay method and zero patient using sublay method (*P* = 0.923, *I*2 = 0%), and OR 2.415, 95% CI 0.661, 8.822 and no statistically significance.

Regarding operative time, eight included studies described operative time using onlay versus sublay. Demetrashvili et al. [17] stated 124 minutes mean time with standard deviation 34 using onlay mesh and 155 minutes with standard deviation 42 using sublay mesh. Ahmed et al. [18] stated 110 minutes mean time with standard deviation 30 using onlay mesh and 80 minutes with standard deviation 32 using sublay mesh. Somooro et al. [11] stated 120 minutes mean time with standard deviation 26 using onlay mesh and 100 minutes with standard deviation 30 using sublay mesh, and Barış Sevinç et al. [19] stated 56 minutes mean time with standard deviation 7 using onlay mesh and 73 minutes with standard deviation 17 using sublay mesh. Deen et al. stated 83 minutes mean time with standard deviation 10 using onlay mesh and 89 minutes with standard deviation 7 using sublay mesh. A. Iljin et al. [16] stated 105 minutes mean time with standard deviation 30 using onlay mesh and 180 minutes with standard deviation 30 using sublay mesh (*P* = 0.001, *I*2 = 95.1%) and OR 12.022, 95% CI − 31,460, 5.616, and there is statistically significance.

Regarding hospital stay, seven included studies described hospital stay using onlay versus sublay. Demetrashvili et al. stated 5.5 mean time with standard deviation 2.5 using onlay mesh and 5 mean with standard deviation 2.5 using sublay mesh. Ahmed et al. [18] stated 8 days mean time with standard deviation 4 using onlay mesh and 4 mean time with standard deviation 2 using sublay mesh. Saeed et al. [12] stated 2 days mean time with standard deviation 0.8 using onlay mesh and 3.9 mean time with standard deviation 1.9 using sublay mesh. Barış Sevinç et al. [19] stated 3.3 mean time with standard deviation 1.9 using onlay mesh and 3.5 mean with standard deviation 2.56 using sublay mesh, and Badawy et al. [13]stated 4.3 mean time with standard deviation 3.7 using onlay mesh and 3.6 mean with standard deviation 2 using sublay mesh. Deen et al. [15] stated 4.6 mean time with standard deviation 0.3 using onlay mesh and 2.6 meantime with standard deviation 0.7 using sublay mesh, and A. Iljin et al. [16]. stated 5 meantime with standard deviation 3 using onlay mesh and 8.5 mean time with standard deviation 3 using sublay mesh (*P* = 0.001, *I*2 = 96.03%) and OR 2.726, 95% CI 1.250, 1.759 with statistically significance.

## Conclusion

According to our results, there is a statistical difference between onlay and sublay regarding intra-operative time as sublay mesh is more time-consuming. Regarding post-operative complications, there is no statistical difference in recurrence, seroma, hematoma, flap necrosis, and infection, but there is a statistical difference regarding in hospital stay as patients with sublay repair stays less than onlay.

## Data Availability

The datasets used and/or analyzed during the current study are available from the corresponding author on reasonable request.
